# Psychosomatic symptoms according to psychiatric diagnosis

**DOI:** 10.1192/j.eurpsy.2023.667

**Published:** 2023-07-19

**Authors:** R. Fernández Fernández, L. Fontecha Banegas, C. Suárez Pérez, D. Gómez Olmeda, I. D. L. M. Santos Carrasco

**Affiliations:** Psychiatry, Hospital Universitario Infanta Cristina, Parla, Spain

## Abstract

**Introduction:**

Psychosomatic symptoms are an important problem that is frequently presented in medical consultations. These symptoms are often associated with psychiatric disorders, especially depressive and anxiety disorders.

**Objectives:**

To study the association between anxiety disorders and psychosomatic symptoms in a sample of patients referred for pathology of functional origin.

**Methods:**

We made a descriptive retrospective study through the use of electronic medical records. The symptom onset and diagnosis were obtained for all patients referred to outpatients for psychosomatic symptoms during a 1-year period. We performed χ² Tests to assess the association of the diagnosis with the occurrence of psychosomatic symptoms.

**Results:**

The only diagnosis that presented statistically significant association was anxiety disorder (χ² = 11.1; p<0.001).

**Table:** 

Anxiety disorder	Psychosomatic symptoms	No	Si	Total
No	Observed	312	7	319
	Expected	306	13.47	319
Yes	Observed	119	12	131
	Expected	125	5.53	131
Total	Observed	431	19	450
	Expected	431	19	450

**Conclusions:**

Our study finds results that follow the line of other studies that show this association, such as Campo’s study which finds that functional somatic symptoms are consistently associated cross-sectionally with anxiety and depressive symptoms (Campo, 2012) or Imran’s study which finds that higher levels of somatization independently and significantly predicted higher anxiety (β=.37, p=.0001) (Imran et al., 2013). However, our results show no association with depressive disorders whereas frequent associations are found in the literature; for example, a recent meta-analysis found that neuroticism and depression had the strongest influence on the association of medically unexplained physical symptoms and frequent healthcare use (den Boeft et al., 2016). This lack of association is probably due to greater ease in identifying depressive disorders as the main pathology versus anxiety disorders.

**References:**

Campo J. V. (2012). Annual research review: functional somatic symptoms and associated anxiety and depression--developmental psychopathology in pediatric practice. Journal of child psychology and psychiatry, and allied disciplines, 53(5), 575–592. den Boeft, M., Twisk, J. W., Terluin, B., Penninx, B. W., van Marwijk, H. W., Numans, M. E., van der Wouden, J. C., & van der Horst, H. E. (2016). The association between medically unexplained physical symptoms and health care use over two years and the influence of depressive and anxiety disorders and personality traits: a longitudinal study. BMC health services research, 16, 100

Imran, N., Ani, C., Mahmood, Z., Hassan, K. A., & Bhatti, M. R. (2014). Anxiety and depression predicted by medically unexplained symptoms in Pakistani children: a case-control study. Journal of psychosomatic research, 76(2), 105–112.

**Disclosure of Interest:**

None Declared

